# Plasma biomarkers of neurodegeneration and neuroinflammation in intracranial atherosclerotic disease

**DOI:** 10.1093/braincomms/fcag125

**Published:** 2026-04-07

**Authors:** Caiyan Liu, Anqi Cheng, Yinxi Zou, Hebo Wang, Zhibing Ai, Shiwen Wu, Linwen Liu, Mingli Li, Yiyang Liu, Zijue Wang, Qianqian Si, Huanyu Zhou, Luke W Bonham, Jennifer S Yokoyama, Weihai Xu, multicenter cohort study ‘stroke incidence and cognitive outcomes of intracranial atherosclerotic stenosis’ (SICO-ICAS) group, Zifan Liu, Shuai Jiang, Hongwei An, Pengfei Wang, Yonggang Hao

**Affiliations:** Department of Neurology, State Key Laboratory of Complex Severe and Rare Diseases, Peking Union Medical College Hospital, Chinese Academy of Medical Sciences and Peking Union Medical College, Beijing 100730, China; Department of Neurology, State Key Laboratory of Complex Severe and Rare Diseases, Peking Union Medical College Hospital, Chinese Academy of Medical Sciences and Peking Union Medical College, Beijing 100730, China; Department of Neurology, State Key Laboratory of Complex Severe and Rare Diseases, Peking Union Medical College Hospital, Chinese Academy of Medical Sciences and Peking Union Medical College, Beijing 100730, China; Department of Neurology, Hebei General Hospital, Shijiazhuang, Hebei 050051, China; Department of Neurology, Shiyan Taihe Hospital, Shiyan, Hubei Province 442000, China; Department of Neurology, First Medical Center of Chinese PLA General Hospital, Beijing 100853, China; Theranostics and Translational Research Center, Institute of Clinical Medicine, Peking Union Medical College Hospital, Chinese Academy of Medical Sciences and Peking Union Medical College, Beijing 100730, China; Department of Radiology, Peking Union Medical College Hospital, Chinese Academy of Medical Sciences and Peking Union Medical College, Beijing 100730, China; Department of Neurology, State Key Laboratory of Complex Severe and Rare Diseases, Peking Union Medical College Hospital, Chinese Academy of Medical Sciences and Peking Union Medical College, Beijing 100730, China; Department of Neurology, State Key Laboratory of Complex Severe and Rare Diseases, Peking Union Medical College Hospital, Chinese Academy of Medical Sciences and Peking Union Medical College, Beijing 100730, China; Department of Neurology, The First Affiliated Hospital with Nanjing Medical University, Nanjing 210029, China; Department of Neurology, State Key Laboratory of Complex Severe and Rare Diseases, Peking Union Medical College Hospital, Chinese Academy of Medical Sciences and Peking Union Medical College, Beijing 100730, China; Department of Radiology and Biomedical Imaging, University of California, San Francisco, San Francisco, CA 94143, USA; Memory and Aging Center, Department of Neurology, Weill Institute for Neurosciences, University of California, San Francisco, San Francisco, CA 94158, USA; Department of Radiology and Biomedical Imaging, University of California, San Francisco, San Francisco, CA 94143, USA; Memory and Aging Center, Department of Neurology, Weill Institute for Neurosciences, University of California, San Francisco, San Francisco, CA 94158, USA; Global Brain Health Institute, University of California San Francisco, San Francisco, CA 94133, USA; Department of Neurology, State Key Laboratory of Complex Severe and Rare Diseases, Peking Union Medical College Hospital, Chinese Academy of Medical Sciences and Peking Union Medical College, Beijing 100730, China

**Keywords:** intracranial atherosclerosis, cognitive impairment, plasma biomarkers, neurodegeneration, neuroinflammation

## Abstract

Intracranial atherosclerotic stenosis is a leading cause of stroke and an increasingly recognized contributor to cognitive impairment and dementia. The mechanisms underlying cognitive dysfunction in intracranial atherosclerotic stenosis remain poorly understood, particularly whether they involve direct vascular brain injury or accelerate neurodegeneration and neuroinflammation. Ultrasensitive plasma biomarkers, including the amyloid-β 42/40 ratio, phosphorylated tau at threonine 217, glial fibrillary acidic protein and neurofilament light chain, offer insights into these processes, but their role in intracranial atherosclerotic stenosis has not been elucidated. We recruited 403 patients with intracranial atherosclerotic stenosis (84 with <50% asymptomatic stenosis, 154 with ≥50% asymptomatic stenosis and 165 with ≥50% symptomatic stenosis) and 98 intracranial atherosclerotic stenosis-free controls across nine centres. Plasma biomarkers were quantified using single-molecule array technology. Cognitive function was assessed with a standardized neuropsychological battery, and multimodal MRI was performed to evaluate infarcts and cerebral small vessel disease burden. Multivariable models examined associations among intracranial atherosclerotic stenosis severity, biomarkers levels, and cognitive impairment. Plasma biomarker levels in patients with asymptomatic stenosis <50% were comparable to those in controls. Glial fibrillary acidic protein was elevated in asymptomatic stenosis ≥50%, while both glial fibrillary acidic protein and neurofilament light chain were increased in symptomatic stenosis. The amyloid-β 42/40 ratio was reduced in symptomatic stenosis ≥50%, whereas phosphorylated tau at threonine 217 was unchanged across groups. In multivariable analyses, none of the plasma biomarkers were significantly associated with cognitive impairment. In contrast, intracranial atherosclerotic stenosis itself was independently associated with cognitive impairment in a graded manner (asymptomatic stenosis <50%: OR = 2.48; asymptomatic stenosis ≥50%: OR = 2.65; symptomatic stenosis: OR = 4.14). These findings indicate that although plasma biomarkers of neurodegeneration and neuroinflammation are altered in advanced intracranial atherosclerotic stenosis, they do not explain the associated cognitive impairment. Instead, cognitive deficits appear to be driven primarily through vascular mechanisms. Our results support reconceptualizing intracranial atherosclerotic stenosis not only as a stroke-prone vascular disorder but also as a covert threat to brain health and cognition. Early identification and aggressive management of intracranial atherosclerotic stenosis, even before conventionally ‘significant’ stenosis or symptoms, are warranted.

## Teaser

Liu *et al*. report that intracranial atherosclerosis (ICAS) severity is independently associated with cognitive impairment, whereas plasma biomarkers of neurodegeneration and neuroinflammation show limited explanatory value. Although biomarker levels change in advanced disease, cognitive deficits appear primarily driven by vascular mechanisms, highlighting ICAS as a covert threat to brain health.

## Graphical Abstract

**Graphical Abstract:**
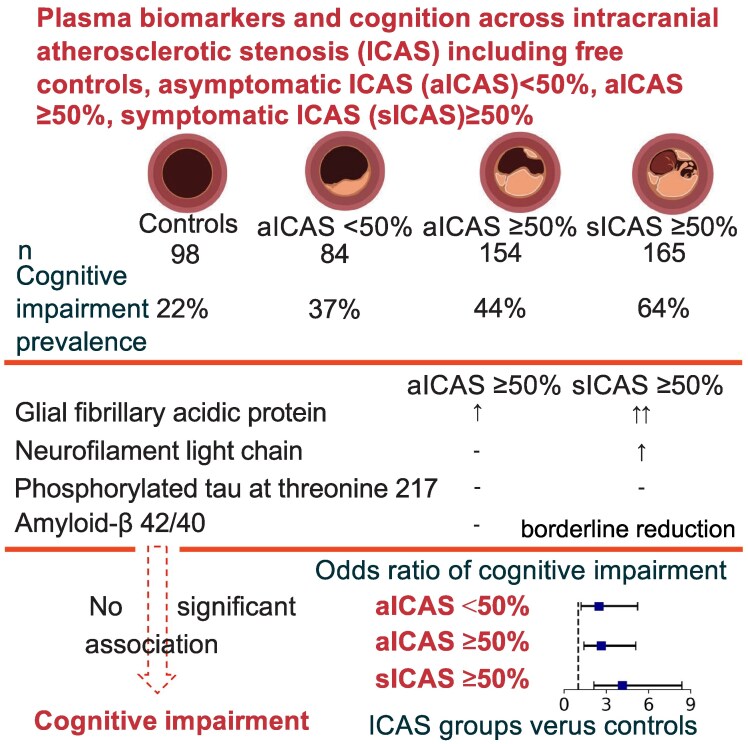

## Introduction

Intracranial atherosclerosis (ICAS) is a leading cause of stroke worldwide and is increasingly recognized as a contributor to cognitive impairment and dementia.^1-6^ Community-based studies indicate that ICAS of varying severity affects 35–45% of older adults and is independently associated with nearly a 50% higher incidence of dementia.^7,8^ The considerable public health burden posed by ICAS-related cognitive impairment underscores the need to clarify its underlying mechanisms and to inform targeted interventions.

ICAS represents a continuum of disease severity, ranging from subclinical plaque formation to flow-limiting stenosis and ultimately ischaemic stroke.^9^ Beyond its well-established role in thromboembolic events, ICAS may contribute to cognitive dysfunction through mechanisms such as chronic progressive hypoperfusion and blood–brain barrier disruption, which can damage vulnerable cognitive networks.^10,11^ A pivotal unresolved question, however, is whether ICAS contributes to cognitive impairment mainly through direct vascular brain injury or by accelerating neurodegeneration and neuroinflammation processes. Although autopsy studies frequently demonstrate co-occurrence of ICAS and Alzheimer’s disease pathology,^12-14^ Positron emission tomography imaging studies in asymptomatic ICAS patients have not consistently shown increased cerebral β-amyloid deposition,^15^ pointing to a more complex relationship.

Recent advances in ultrasensitive plasma biomarker assays now permit *in vivo* quantification of proteins that reflect neurodegeneration and neuroinflammation, offering new opportunities to disentangle vascular contributions to cognitive impairment.^16-18^ The amyloid-β 42/40 (Aβ42/40) ratio and phosphorylated tau at threonine 217 (pTau217) are established plasma correlates of Alzheimer’s disease pathology, whereas glial fibrillary acidic protein (GFAP) reflects astrocytic activation and neurofilament light chain (NfL) reflects axonal injury.^19^ These biomarkers have shown robust associations with cognitive decline, dementia progression and cerebrovascular pathology in diverse populations. However, their role in ICAS remains largely unexplored. Clarifying whether ICAS-related cognitive impairment is related to measurable changes in these circulating biomarkers is therefore of both mechanistic and translational importance.

In this multicentre, cross-sectional study, participants were stratified into four groups: ICAS-free controls, asymptomatic ICAS (aICAS) with <50% stenosis, asymptomatic ICAS with ≥50% stenosis and symptomatic ICAS (sICAS ≥50%, defined as ≥50% stenosis with stroke or transient ischaemic attack). This classification system captures biologically and clinically meaningful categories of ICAS severity. In this context, ≥50% stenosis is used to denote haemodynamic significance, reflecting a degree of arterial narrowing associated with compromised cerebral blood flow, whereas symptomatic status is used as a clinical indicator of plaque instability, defined by the occurrence of recent ICAS-related ischaemic events. This stage-based framework provided a biologically grounded context in which to examine whether different pathological processes contribute to cognitive impairment across the ICAS spectrum. On the basis of prior literature, we therefore posited that ICAS-related cognitive impairment may be differentially associated with circulating biomarkers reflecting distinct biological processes, including Alzheimer’s disease–related pathology, neurodegeneration and neuroinflammation. Accordingly, within this stage-based ICAS framework, we investigated whether biomarkers indexing these processes show distinct patterns of association with ICAS severity and cognitive status.

## Materials and methods

This study follows the Strengthening the Reporting of Observational Studies in Epidemiology (STROBE) guidelines.^20^

### Study design

This cross-sectional analysis was based on baseline data from an ongoing prospective multicentre cohort study.^21^ Participants with aICAS (without a history of ischaemic stroke or transient ischaemic attack), sICAS (with a history of ischaemic stroke or transient ischaemic attack) and age- and sex-matched ICAS-free controls were enrolled.

### Participant recruitment

Inclusion criteria of ICAS patients were: (i) aged ≥35 years; (ii) radiologically confirmed ICAS in branches of the Circle of Willis (intracranial internal carotid, middle cerebral, anterior cerebral, posterior cerebral, basilar, or vertebral arteries) verified by magnetic resonance angiography and high-resolution vessel wall imaging; (iii) functional independence (modified Rankin Scale ≤2); (iv) Stroke-related criteria differed by subgroup: no history of ischaemic stroke for aICAS, and no ischaemic stroke within 3 months prior to enrolment for sICAS. Exclusion criteria were: (i) extracranial stenosis (≥50%); (ii) non-atherosclerotic intracranial stenosis (e.g. vasculitis, dissection and moyamoya disease); (iii) history of neurodegenerative diseases (Alzheimer’s disease, Lewy body dementia, frontotemporal dementia, Parkinson’s disease); (iv) conditions affecting cognition or plasma biomarker validity, including cognitive comorbidities (traumatic brain injury, central nervous system infections, metabolic encephalopathy), factors influencing Alzheimer’s disease biomarker metabolism (e.g. renal dysfunction) or life-limiting conditions (e.g. advanced malignancies); and (v) incomplete clinical, imaging or biomarker data. ICAS-free controls were recruited from community-dwelling adults and health screening centres; eligibility required no history of stroke or transient ischaemic attack, no significant intracranial or extracranial stenosis and no known neurological disorders affecting cognition.

### Clinical data acquisition

Standardized case report forms collected: (i) demographics [age, sex, educational attainment (years) and body mass index]; (ii) medical history (hypertension, diabetes mellitus, hyperlipidaemia, prior stroke or transient ischaemic attack, thyroid disorders, autoimmune diseases and malignancies); (iii) family history of cerebrovascular disease and dementia (in first-degree relatives); (iv) medications (use of antithrombotic agents, antihypertensives and lipid-lowering agents); (v) laboratory workup (fasting glucose, lipid panel and apolipoprotein E [*APOE* ε4 allele] genotyping); (vi) cardiovascular assessments (blood pressure measurements, carotid duplex ultrasound findings and echocardiography results).

### Plasma biomarker assessment

Venous blood samples were collected in EDTA anticoagulant tubes, processed within 2 h and stored at −80°C. Plasma concentrations of Aβ40, Aβ42, NfL, GFAP and pTau217 were measured using ultra-sensitive Simoa technology (Quanterix, MA, USA) on an HD-X platform (GBIO, Hangzhou, China) following the manufacturer’s protocols. The Neurology 4-Plex E (Cat. Nos. 503864 and 504166) and ALZpath pTau217 (Cat No.: 999036) assay kits were used. All operators were blinded to participants’ clinical status.

### Neuropsychological assessment

Participants underwent a comprehensive neuropsychological battery administered by board-certified neuropsychologists. Global cognition was assessed using the Mini-Mental State Examination (MMSE) and Montreal Cognitive Assessment (MoCA). Functional status was evaluated with the activities of daily living scale, and psychiatric symptoms were assessed using the Hamilton Anxiety and Depression Rating Scales.

For the purposes of this study, cognition was operationalized into five domains based on standard neuropsychological measures: memory (auditory verbal learning test, paired association learning test and Rey–Osterrieth complex figure recall); attention/executive function (trail making tests, digit span, clock drawing test and symbol digit modalities test); language (Boston naming test and verbal fluency); visuospatial ability (Rey–Osterrieth complex figure copy); and calculation ability, indexed by performance on the Arithmetic subtest of the Wechsler Adult Intelligence Scale (WAIS), which assesses mental arithmetic and numerical processing. Cognitive status [cognitively normal, mild cognitive impairment (MCI), dementia] was adjudicated by two independent cognitive neurologists using the criteria outlined in the Diagnostic and Statistical Manual of Mental Disorders, Fifth Edition (DSM-5) and current vascular cognitive impairment diagnostic guidelines.^22-24^ Cognitively normal status was defined as having normal performance on neuropsychological testing and preserved activities of daily living. MCI was defined as impairment in at least one cognitive domain, as indicated by established neuropsychological test performance thresholds according to guideline recommendations, combined with evidence of subtle functional decline. Dementia was characterized by impairment in at least one cognitive domain, accompanied by significant functional decline. Cognitive impairment was categorized as either MCI or dementia.

### MRI assessment

All participants underwent a standardized multimodal MRI protocol, including conventional cranial MRI, and three-dimensional time-of-flight magnetic resonance angiography (Supplementary Method 1). The degree of ICAS stenosis (<50%, or ≥50%) was quantified using criteria from the Warfarin-Aspirin Symptomatic Intracranial Disease trial.^25^ Participants were classified into (i) ICAS-free controls; (ii) aICAS with <50% stenosis; (iii) aICAS with ≥50% stenosis; (iv) sICAS with ≥50% stenosis. White matter hyperintensity burden and lacunar infarcts were evaluated on axial T2-weighted fluid-attenuated inversion recovery sequences The Fazekas scale was applied to semi-quantify the white matter hyperintensity burden. Cerebral microbleeds were identified and counted using T2* gradient-recalled echo or susceptibility-weighted imaging. Cerebral small vessel disease (CSVD) burden was scored ordinally (0–7 points) using validated criteria, encompassing lacunes, microbleeds and white matter hyperintensities.^26^ MRI-based infarct identification was performed by trained raters using a standardized visual rating protocol based on predefined anatomical regions. The following predefined anatomical regions were systematically evaluated for infarcts: the basal ganglia and adjacent white matter, angular gyrus, thalamus, basal forebrain, corpus callosum, anterior cingulate cortex and vascular territory–based regions including the anterior cerebral artery and posterior cerebral artery territories. Infarcts in these cognitively relevant regions were recorded to quantify the proportion of ICAS participants with imaging-visible infarcts. These regional infarct indicators were not used to define MRI rating scores or to assign participants to the main ICAS groups but were included in sensitivity analyses to evaluate the robustness of the primary results.

All MRI markers of interest were identified by manual visual inspection, without any automated or quantitative post-processing prior to image review. Image review and rating were performed independently by two trained raters according to predefined criteria (Supplementary Method 2), with interrater Cohen’s *κ* values reported in the Supplementary Table 1. Following independent review, discrepancies between raters were resolved through consensus discussion, and the consensus ratings were used for all subsequent analyses.

### Statistical analysis

Baseline characteristics were summarized as means (standard deviation) for continuous variables and proportions for categorical variables. To characterize group-level differences in demographic and clinical features, demographic characteristics, vascular risk factors and CSVD burden were compared across four ICAS groups using unadjusted group comparisons, with overall differences assessed by the Kruskal–Wallis test for continuous variables and the *χ*^2^ test for the categorical variables, as appropriate. Neuropsychological test performance was compared across ICAS groups using analysis of covariance (ANCOVA), adjusting for age, sex and years of education; prespecified pairwise comparisons were performed using the same adjusted models.

### Associations between ICAS severity and plasma biomarkers

To examine whether plasma biomarkers representing different aspects of brain health varied with ICAS severity (grouped as above), linear regression models were used to assess associations between ICAS severity and plasma biomarker levels adjusting for age, sex, *APOE* ε4 carrier status. Biomarkers included the natural log-transformed plasma Aβ42/40 ratio, pTau217, GFAP and NfL.

### Associations of ICAS severity and plasma biomarkers with cognitive impairment

To examine the associations of ICAS severity and plasma biomarkers with cognitive impairment, multivariable logistic regression models were constructed with cognitive impairment [classified as cognitively normal versus any impairment (MCI or dementia)] as the dependent variable, using the ICAS-free control group as the reference category.

Model A included ICAS severity as the independent variable, adjusting for age, sex, education years, study centre, hypertension, diabetes mellitus, smoking status and *APOE* ε4 carrier status. Model B additionally incorporated each plasma biomarker separately (ln-transformed plasma Aβ42/40 ratio, pTau217, GFAP or NfL) alongside the ICAS severity, while retaining the same covariate adjustments as in Model A. Model C simultaneously included ICAS severity, all four plasma biomarkers (ln-transformed Aβ42/40 ratio, pTau217, GFAP and NfL), and CSVD burden as independent variables, with covariate adjustments identical to those in Model A. Odds ratios (ORs) and 95% confidence intervals (CIs) were reported.

### Sensitivity analyses

Prespecified sensitivity analyses were conducted to assess the robustness of the primary findings and to explore potential effect modification by age and sex, as well as the influence of covert cerebrovascular injury; these analyses were not used to define the primary analytic groups or conclusions. Sensitivity analyses included: (i) stratification by age (<60 versus ≥ 60 years) and sex; (ii) exclusion of participants with silent MRI infarcts from the control, aICAS <50%, and aICAS ≥50% groups while retaining primary covariates and (iii) age-stratified analyses (<60 and ≥60 years) in which separate linear regression models were fitted within each subgroup to assess associations between ICAS severity and plasma biomarkers, adjusting for age, sex and *APOE* ε4 carrier status.

Analyses were conducted using IBM SPSS Statistics (Version 26.0). All tests were two-sided, and a uniform nominal significance threshold of *P* < 0.05 was used across all analyses; no adjustment for multiple comparisons was applied. Given the exploratory nature of the study, findings should be interpreted as hypothesis-generating.

### Standard protocol approvals, registrations and patient consents

The study was approved by the Institutional Review Board of Peking Union Medical College Hospital, Chinese Academy of Medical Sciences (IRB# JS-3479D; 14 April 2022), and adhered to the Declaration of Helsinki. Written informed consent was obtained from all participants.

## Results

Of 503 screened participants across nine sites (Supplementary Table 2), 403 ICAS patients (84 aICAS <50%, 154 aICAS ≥50%, 165 sICAS ≥50%) and 98 ICAS-free controls were included after exclusions (*n* = 100; Supplementary Fig. 1). Age distributions were similar across groups, whereas the proportion of men was significantly higher in the sICAS ≥50% group (Table 1). Compared with controls, participants with sICAS ≥50% had significantly fewer years of education and a higher prevalence of hypertension, diabetes and current smoking. CSVD burden increased stepwise with increasing ICAS severity (Table 1, Supplementary Tables 3 and 4). The prevalence of cognitive impairment (MCI or dementia) differed significantly across groups, increasing from 22% in controls to 37% in aICAS <50%, 43% in aICAS ≥50%, and 63% in sICAS ≥50% (Table 1). Performance on global cognitive measures (MMSE and MoCA) and all neuropsychological tests declined progressively with increasing ICAS severity (Table 2). Pairwise comparisons versus controls on the neuropsychological performance are reported in Supplementary Table 5.

**Table 1:** Demographic factors and clinical data of participants

	Group				
	Controls	aICAS <50%	aICAS ≥50%	sICAS ≥50%	P1
No.	**98**	**84**	**154**	**165**	
Demographic factors					
Age, mean (SD), y	60.7(9.1)	57.7 (11.8)	58.8 (11.2)	61.3 (10.0)	0.079
Male, No. (%)	45 (46%)	42 (50%)	73 (47%)	109 (66%)	0.001
Education, mean (SD), y	12.61 (4.55)	13.10 (4.68)	12.00 (4.36)	10.62 (4.00)	<0.001
Hypertension, No. (%)	32 (33%)	38 (45%)	85 (55%)	107 (65%)	<0.001
Diabetes mellitus, No. (%)	17 (17%)	21 (25%)	36 (23%)	65 (39%)	<0.001
Hyperlipidemia, No. (%)	46 (47%)	49 (58%)	95 (62%)	103 (62%)	0.069
Atrial fibrillation, No. (%)	1 (1.0%)	1 (1.2%)	1 (0.6%)	1 (0.6%)	0.951
Coronary disease, No. (%)	9 (9.2%)	8 (9.5%)	15 (9.7%)	22 (13%)	0.639
Smoking, No. (%)	24 (24%)	18 (21%)	37 (24%)	66 (40%)*	0.002
Alcohol taken, No. (%)	50 (51%)	39 (46%)	69 (45%)	85 (52%)	0.607
APOE ε4 carrier, No. (%)	18 (18%)	7 (8.3%)	28 (18%)	30 (18%)	0.175
ICAS characteristics					
Multiple vessels involved, No. (%)	0 (0%)	41 (49%)	103 (67%)	127 (77%)	NA
Posterior circulation involved, No. (%)	0 (0%)	32 (38%)	75 (49%)	103 (62%)	NA
Visible cerebral infarction lesions on MRI, No. (%)	7 (7.1%)	13 (15%)	29 (19%)	132 (80%)	<0.001
CSVD burden, mean (SD)	0.96 (1.05)	1.35 (1.52)	1.41 (1.48)	2.63 (1.87)	<0.001
Cognitive status					
MCI, No. (%)	22 (22%)	29 (35%)	59 (38%)	73 (44%)	<0.001
Dementia, No. (%)	0 (0%)	2 (2.4%)	8 (5.2%)	32 (19%)	<0.001
Any cognitive impairment, No. (%)	22 (22%)	31 (37%)	67 (43%)	105 (63%)	<0.001

Values are presented as mean ± SD, median (IQR), or *n* (%), as appropriate. P1 indicates the overall comparison across the four groups (Kruskal–Wallis test for continuous variables; *χ*^2^ test for categorical variables). For ICAS-specific characteristics (e.g. multiple vessels involved; posterior circulation involved), P1 is not applicable (NA) because these variables are defined only among participants with ICAS. Pairwise comparisons versus controls are reported in Supplementary Table 3. All tests were two-sided; nominal *P* < 0.05.

Abbreviations: ICAS, intracranial atherosclerosis; controls, ICAS-free controls; aICAS <50%, asymptomatic intracranial atherosclerosis with <50% stenosis; aICAS ≥50%, asymptomatic ICAS with ≥50% stenosis; sICAS ≥50%, symptomatic ICAS with ≥50% stenosis; CSVD, cerebral small vascular disease; SD, standard deviations; MCI, mild cognitive impairment.

**Table 2:** The neuropsychological test performance of participants

	Group				
	Controls	aICAS <50%	aICAS ≥50%	sICAS ≥50%	P1
**No.**	98	84	154	165	
**MMSE, mean (SD)**	27.35 (1.91)	26.71 (3.42)	26.34 (3.06)	25.12 (4.29)	<0.001
**MOCA, mean (SD)**	24.92 (3.10)	24.23 (4.16)	23.44 (4.38)	21.38 (5.41)	<0.001
**Auditory verbal learning test, total, mean (SD)**	29.77 (8.93)	28.44 (11.00)	26.76 (11.49)	21.77 (11.11)	<0.001
**Paired association learning test, mean (SD)**	12.19 (5.00)	11.40 (5.74)	10.10 (5.34)	9.05 (5.21)	0.002
**Rey complex figure recall, mean (SD)**	20.48 (7.73)	19.09 (9.41	17.89 (8.55)	14.68 (9.07)	<0.001
**Digit span-forward, mean (SD)**	7.78 (1.54)	8.12 (1.23)	7.87 (1.51)	7.12 (1.53)	0.002
**Digit span-backward, mean (SD)**	4.82 (1.55)	5.05 (1.71)	4.93 (1.60)	4.16 (1.58)	0.022
**Trail making Test-A, mean (SD), seconds**	64.98 (49.31)	59.72 (29.04)	59.66 (30.97)	76.78 (50.81)	0.053
**Trail making Test-B, mean (SD), seconds**	146.10 (58.32)	159.11 (71.33)	165.34 (84.75)	183.08 (92.62)	0.014
**Clock drawing test, mean (SD)**	2.68 (0.57)	2.61 (0.64)	2.58 (0.68)	2.24 (0.88)	<0.001
**Symbol digit modalities test, mean (SD)**	42.06 (14.66)	42.22 (16.84)	41.27 (16.26)	31.11 (16.84)	<0.001
**Rey complex figure copy, mean (SD)**	34.22 (4.00)	33.94 (4.86)	33.21 (6.02)	30.97 (8.31)	0.017
**Boston naming test, mean (SD)**	26.09 (3.04)	25.46 (4.08)	24.68 (3.93)	23.98 (4.47)	0.009
**Verbal fluency, naming animal, mean (SD)**	19.83 (5.07)	20.11 (7.83)	18.84 (6.70)	15.30 (6.40)	<0.001
**Calculation, mean (SD)**	11.82 (3.73)	11.17 (3.73)	10.47 (3.72)	9.55 (3.83)	<0.001

*P*-value: P1 denotes the overall between-group comparison using ANCOVA adjusting for age, sex and education. Pairwise comparisons versus controls are reported in Supplementary Table 5. All tests were two-sided; nominal *P* < 0.05. Abbreviations: ICAS, intracranial atherosclerosis; aICAS, asymptomatic ICAS; sICAS, symptomatic ICAS; SD, standard deviation.

### Associations between ICAS severity and plasma biomarkers

**Figure 1:**
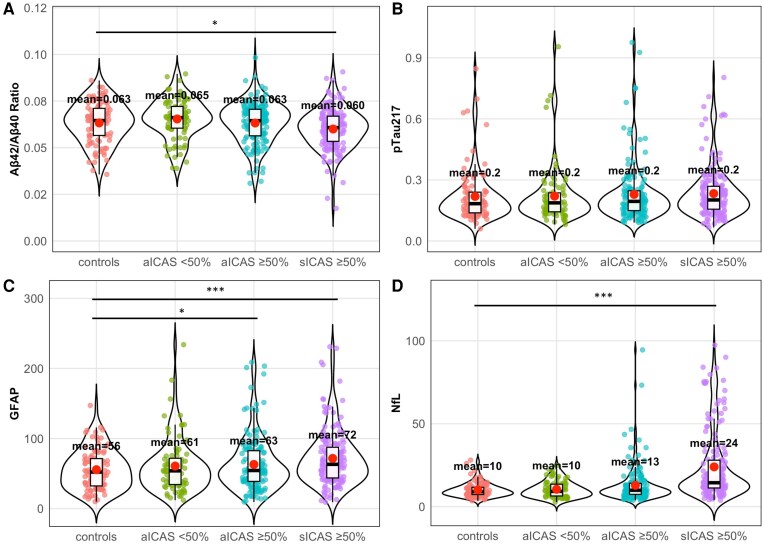
**Plasma biomarkers by intracranial atherosclerosis (ICAS) severity.** Plasma biomarker levels are shown for four groups: ICAS-free controls, asymptomatic ICAS with <50% stenosis (aICAS < 50%), asymptomatic ICAS with ≥50% stenosis (aICAS ≥ 50%) and symptomatic ICAS with ≥50% stenosis (sICAS ≥ 50%). Sample sizes were *n* = 98, *n* = 84, *n* = 154 and *n* = 165, respectively. (**A**) Aβ42/Aβ40 ratio. (**B**) pTau217. (**C**) GFAP. (**D**) NfL. Statistical analysis. For each biomarker, associations with ICAS severity (categorical; control group as reference) were tested using multivariable linear regression, adjusting for age, sex and *APOE* ε4 carrier status (dependent variables: ln-transformed Aβ42/40 ratio, pTau217, GFAP and NfL). Results are reported as adjusted regression coefficients (*β*) with 95% confidence intervals (CI), together with the corresponding *t*-statistic and two-sided *P* value for each contrast versus controls. Significant contrasts were: GFAP—aICAS ≥ 50% versus controls, *β* = 10.69 (95% CI 2.31–19.06), *t* = 2.51, *P* = 0.012; sICAS ≥ 50% versus controls, *β* = 17.40 (95% CI 9.06–25.73), *t* = 4.10, *P* < 0.001. NfL—sICAS ≥ 50% versus controls, *β* = 13.59 (95% CI 10.15–17.03), *t* = 7.76, *P* < 0.001. Aβ42/40 ratio—sICAS ≥ 50% versus controls, *β* = −0.003 (95% CI −0.006 to 0.000), *t* = −2.28, *P* = 0.023. All other contrasts were non-significant (*P* ≥ 0.05). Experimental unit. The experimental unit was the individual participant (one plasma sample per participant; no repeated biological replicates). All groups had *n* > 10; therefore, a requirement to display individual data points based on a small sample size did not apply. Asterisks indicate two-sided *P* < 0.05. Abbreviations: ICAS, intracranial atherosclerosis; aICAS, asymptomatic ICAS; sICAS, symptomatic ICAS; Aβ, amyloid-β; pTau217, phosphorylated tau (threonine 217); GFAP, glial fibrillary acidic protein; NfL, neurofilament light chain.

Plasma biomarker levels in patients with aICAS <50% were comparable to those in controls. GFAP was elevated in aICAS ≥50% (*β* = 10.69, 95% CI [2.31, 19.06], *t* = 2.51, *P* = 0.012). In sICAS ≥50%, both GFAP (*β* = 17.40, 95% CI [9.06, 25.73], *t* = 4.10, *P* < 0.001) and NfL (*β* = 13.59, 95% CI [10.15, 17.03], *t* = 7.76, *P* < 0.001) were elevated; the Aβ42/40 ratio showed a reduction (*β* = −0.003, 95% CI [−0.006, 0.00], *t* = −2.28, *P* = 0.023). pTau217 was unchanged across groups (Fig. 1). All plasma biomarker analysis in ICAS groups was adjusted for age, sex and *APOE* ε4 carrier status.

### Associations of ICAS severity and plasma biomarkers with cognitive impairment

**Figure 2:**
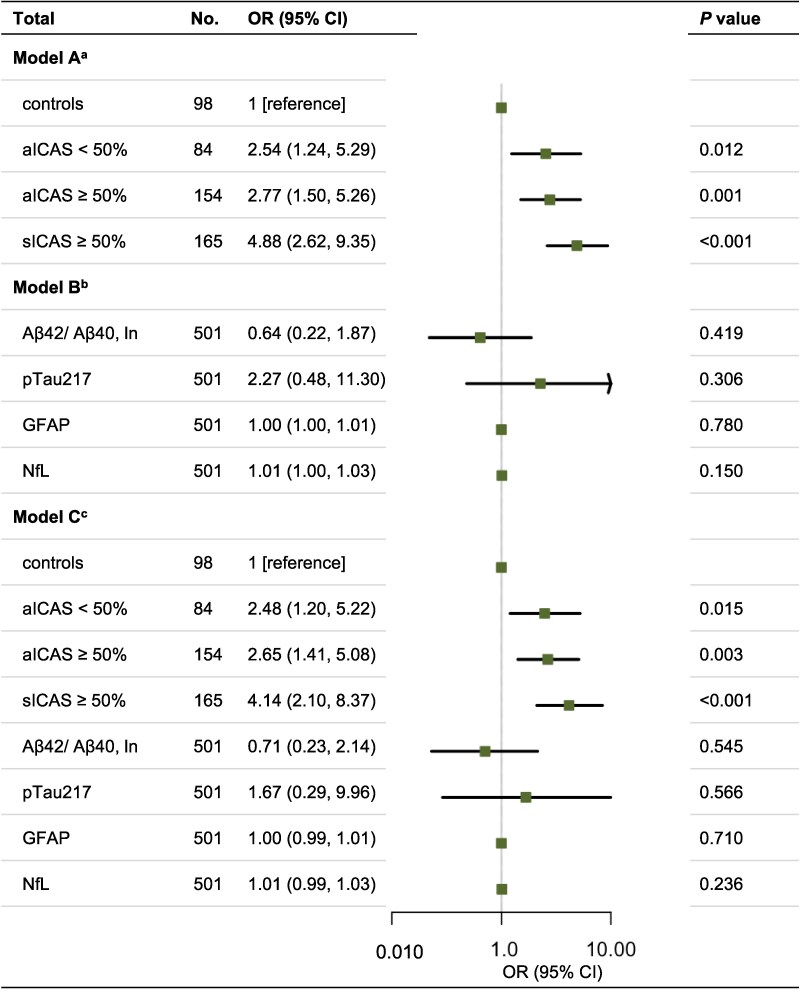
**Associations of ICAS severity and plasma biomarkers with cognitive impairment.** Forest plots show adjusted odds ratios (ORs) with 95% confidence intervals (CIs) from multivariable logistic regression models with cognitive impairment (mild cognitive impairment or dementia versus cognitively normal) as the outcome and the ICAS-free control group as the reference category. Statistical analysis. For each model coefficient, two-sided *P* values were obtained from Wald tests in logistic regression (Wald *χ*^2^ statistics are reported in Supplementary Table 6). A uniform nominal significance threshold of *P* < 0.05 was used across analyses, with no adjustment for multiple comparisons. Experimental unit. The experimental unit was the individual participant (one cognitive outcome classification and one set of biomarker measurements per participant). All groups had *n* > 10; therefore, the requirement to display individual data points based on a small sample size did not apply. Models. Model A included ICAS severity and adjusted for age, sex, years of education, study centre, hypertension, diabetes mellitus, smoking status and *APOE* ε4 carrier status. Model B additionally included each plasma biomarker separately (ln-transformed Aβ42/40 ratio, pTau217, GFAP, or NfL) with the same covariate adjustments as Model A. Model C simultaneously included ICAS severity, all four plasma biomarkers (ln-transformed Aβ42/40 ratio, pTau217, GFAP and NfL), and CSVD burden, with covariate adjustments identical to Model A. Abbreviations: ICAS, intracranial atherosclerosis; aICAS, asymptomatic ICAS; sICAS, symptomatic ICAS; Aβ, amyloid-β; pTau217, phosphorylated tau (threonine 217); GFAP, glial fibrillary acidic protein; NfL, neurofilament light chain; CSVD, cerebral small vessel disease.

In multivariable binary logistic regression models adjusted for age, sex, years of education, hypertension, diabetes, smoking status and *APOE* ε4 carrier status, ICAS was associated with higher odds of cognitive impairment in a graded manner (aICAS <50%: OR = 2.54, 95%CI [1.24, 5.29], *P* = 0.012; aICAS ≥50%: OR = 2.77, 95%CI [1.50, 5.26], *P* = 0.001; sICAS: OR = 4.88, 95%CI [2.62, 9.35], *P* < 0.001). When each plasma biomarker was included separately in the model (Model B), none was significantly associated with cognitive impairment: ln-transformed Aβ42/40 ratio (OR = 0.64, 95%CI [0.22, 1.87], *P* > 0.05), pTau217 (OR = 2.27, 95%CI [0.48, 11.30], *P* > 0.05), GFAP (OR = 1.00, 95%CI [1.00, 1.01], *P* > 0.05) or NfL (OR = 1.01, 95%CI [1.00, 1.03], *P* > 0.05). Model C simultaneously included the ICAS severity, all four plasma biomarkers (ln-transformed Aβ42/40 ratio, p-tau217, GFAP and NfL), and CSVD burden as independent variables, while maintaining the same covariate adjustments. This model yielded consistent results regarding the association between ICAS and cognitive impairment (aICAS <50%: OR = 2.48, 95%CI [1.20, 5.22], *P* = 0.015; aICAS ≥50%: OR = 2.65, 95%CI [1.41, 5.08], *P* = 0.003; sICAS: OR = 4.14 95%CI [2.10, 8.37], *P* < 0.001) (Fig. 2, Supplementary Table 6).

### Sensitivity analyses

Sensitivity analyses yielded results consistent with the primary findings. Stratified analyses by age and sex showed that aICAS remained independently associated with cognitive impairment, whereas no consistent associations were observed for plasma biomarkers (Supplementary Fig. 2). After excluding participants with silent infarcts, the association between aICAS and cognitive impairment persisted, while plasma biomarkers remained non-significant (Supplementary Fig. 3). In age-stratified analyses, separate within-stratum linear regression models (≥60 years and <60 years) comparing each ICAS group with controls showed patterns consistent with the overall findings (Supplementary Fig. 4).

## Discussion

This multicentre cross-sectional study provides evidence that cognitive impairment in ICAS is largely independent of plasma biomarkers traditionally linked to neurodegeneration and neuroinflammation. We observed a strong, graded association between ICAS severity and cognitive dysfunction, evident even at <50% asymptomatic stenosis. Although plasma biomarkers showed distinct changes with disease severity, including elevations in GFAP at ≥50% asymptomatic stenosis and further increases in GFAP and NfL in symptomatic ICAS, these alterations did not account for the observed cognitive deficits in multivariable analyses.

The dissociation between plasma biomarker alterations and cognition provides pathophysiological insight. The early elevation in GFAP in ≥50% stenosis likely reflects astrocytic activation triggered by haemodynamically significant lesions, even before overt ischaemia.^27-29^ The subsequent rise in NfL observed in symptomatic ICAS indicates neuroaxonal injury after clinical ischaemic events.^18,30^ However, the absence of an independent association with cognition suggests that these biomarker changes reflect secondary injury processes rather than primary drivers of cognitive decline. Similarly, the reduction in Aβ42/40 ratio in sICAS, without consistent changes in pTau217, appears more compatible with a downstream consequence of advanced cerebrovascular pathology than with primary Alzheimer’s disease pathology.^18,31-34^ The relative stability of all biomarkers in <50% stenosis, despite measurable cognitive deficits, further supports the view that early ICAS impairs cognition through vascular mechanisms not captured by these circulating markers.

The persistent association between ICAS and cognitive impairment after adjusting for vascular risk factors, *APOE* genotype, CSVD burden and plasma biomarkers underscores the likely direct role of ICAS-specific pathology. Sensitivity analyses excluding silent infarcts and stratification by age and sex confirmed the robustness of this association. Several mechanisms may contribute to ICAS-related cognitive impairment: chronic cerebral hypoperfusion downstream of stenotic vessels, particularly in watershed territories crucial for executive and memory functions, may induce synaptic dysfunction and neuronal loss without producing biomarker elevations^10,35^ disruption of neurovascular unit function and blood–brain barrier integrity may lead to perivascular inflammation and neurotoxic leakage^9^; and microembolism from unstable plaques may cause microinfarcts undetectable by conventional imaging but disruptive to cognitive networks.^36-38^

Our findings have important clinical implications. The association between even <50% asymptomatic stenosis and cognitive impairment suggests that plaque presence alone, potentially via endothelial dysfunction, local vessel wall inflammation, or subtle haemodynamic compromise, may initiate harmful brain processes long before conventional thresholds of ‘significant’ stenosis are met. These findings support earlier recognition and management of ICAS, with strategies aimed not only at preventing stroke but also at preserving cognitive health. Aggressive control of vascular risk factors, lifestyle interventions, and exploration of therapies that stabilize plaques or optimize cerebral perfusion should be considered before the onset of symptomatic disease.^39-42^

Several limitations should be acknowledged. First, as a cross-sectional study, causality cannot be inferred, and longitudinal data are needed to confirm temporal dynamics between ICAS severity, biomarker changes and cognitive decline. Second, plasma biomarkers, although validated surrogates, may not capture all relevant aspects of vascular injury, synaptic dysfunction, or neuroinflammation.^43^ Third, specific vascular factors, such as impaired cerebrovascular reactivity and collateral circulation, were not fully accounted for, and the use of a relatively coarse measure of CSVD burden did not capture the spatial distribution of small vessel disease or the vascular territory of stenosis, which may differentially influence the severity and domain-specific profile of cognitive impairment (e.g. preferential effects on executive and processing speed versus memory domains depending on the affected networks).^44^ Finally, the modest sample size in the <50% stenosis group limited detailed analyses, and recruitment from clinical centres may restrict generalizability to population-based cohorts.

Future research should adopt longitudinal designs to track ICAS progression, biomarker evolution and cognitive trajectories. Broader biomarker panels, including markers of endothelial dysfunction, blood–brain barrier breakdown and synaptic injury, may yield deeper insights.^43^ Advanced neuroimaging (e.g. arterial spin labelling, vessel wall imaging, diffusion tensor imaging) could reveal structural and functional correlates of ICAS-related cognitive decline.^45-48^ Interventional studies should evaluate whether intensive medical therapy or revascularization alters biomarker dynamics and improves cognitive outcomes.

## Conclusion

Our findings support ICAS as a major, independent contributor to cognitive impairment, acting predominantly through vascular mechanisms rather than measurable systemic neurodegeneration or neuroinflammation. While plasma biomarkers such as GFAP and NfL reflect secondary consequences of advanced disease, they do not explain early cognitive deficits that already emerge at subthreshold stenosis. These results underscore the need to reconceptualize ICAS not only as a stroke-prone vascular disorder but also as a covert threat to brain health and cognition. Early identification and aggressive management of ICAS, even before conventionally ‘significant’ stenosis or symptoms, may help preserve cognitive resilience and delay dementia.

## Supplementary Material

fcag125_Supplementary_Data

## Data Availability

Data will be shared on a reasonable request to the corresponding author.

## Appendix

The multicenter cohort study ‘stroke incidence and cognitive outcomes of intracranial atherosclerotic stenosis’ (SICO-ICAS) group coinvestigators: Zifan Liu, Shuai Jiang, Hongwei An, Pengfei Wang, Yonggang Hao.
