# Supplementary material for: Plasma biomarkers of neurodegeneration and neuroinflammation in intracranial atherosclerotic disease
Source: Brain Commun. 2026 Apr 7;8(3):fcag125. doi: 10.1093/braincomms/fcag125 (PMC13180752; doi:10.1093/braincomms/fcag125)
Supplement: fcag125_Supplementary_Data [file fcag125_supplementary_data.pdf]

Supplementary Method 1: Standardized multimodal MRI protocol

Supplementary Method 2: Visual rating of cerebral small vascular disease burden and infarction determination

Supplementary Table 1: Inter-rater agreement between two raters for ICAS and CSVD markers

Supplementary Table 2: Distribution of Enrolled Participants by Study Sites

Supplementary Table 3: Demographic factors and clinical data of participants (Pairwise comparisons versus controls)

Supplementary Table 4: Association of CSVD neuroimaging biomarkers with ICAS continuum

Supplementary Table 5: The neuropsychological test performance of participants (Pairwise comparisons versus controls)

Supplementary Table 6: Multivariable logistic regression analysis of ICAS severity and plasma biomarkers in relation to cognitive impairment

Supplementary Figure 1: Flow diagram of the recruitment of study participants

Supplementary Figure 2: Stratified associations of ICAS with cognitive impairment

Supplementary Figure 3: Association between asymptomatic intracranial atherosclerosis (aICAS) and cognitive impairment after excluding participants with silent infarcts

Supplementary Figure 4: Plasma biomarkers by ICAS severity (stratified analysis)

Supplementary Method 1: Standardized multimodal MRI protocol

All participants were examined on one of four harmonized scanners—GE Discovery MR750 (Peking Union Medical College Hospital, Hebei General Hospital, The General Hospital of the Chinese People's Liberation Army , Dushu Lake Hospital Affiliated to Soochow University), GE premier (West China Hospital), GE 3T PIONEER (Liuzhou Traditional Chinese Medical Hospital), Siemens MAGNETOM Vida (Hebei General Hospital), Siemens MAGNETOM Sempra (Taihe Hospital of Shiyan), Siemens MAGNETOM Skyra (Weihai Municipal Hospital), Siemens MAGNETOM Prisma (Weihai Municipal Hospital), Siemens MAGNETOM Trio (Weihai Municipal Hospital) or Philips Achieva 3.0T TX (The Third Affiliated Hospital of Guangzhou Medical University) —each fitted with 20- to 64-channel head/neck coils and equipped with automatic positioning (AutoAlign/BioMatrix/Smart-Select) to ensure identical cranio-caudal orientation. The core protocol comprised: (1) a 3-slab, magnetization-prepared 3D time-of-flight MRA (nominal 0.7-1.0 mm isotropic at 3 T, 0.9 mm at 1.5 T; TR ≈ 30-40 ms, TE ≈ 5-8 ms, flip 20-25°); (2) sagittal 3D T1-weighted IR-GRE (MPRAGE/BRAVO/uCR; 1.0 mm isotropic; TR ≈ 2100 ms, TI ≈ 1100 ms, TE ≈ 2 ms); (3) axial 2D T2-weighted TSE (4-5 mm, TR ≥ 5000 ms, TE 100-120 ms); (4) axial 2D FLAIR (3 mm, TR ≥ 9000 ms, TE 120 ms, TI 2500 ms); (5) axial 2D T2* GRE (3-4 mm, TR ≈ 620-700 ms, TE 20-25 ms) and 3D SWI/SWAN with flow compensation (in-plane ≤0.7 mm, slice 1.2-1.5 mm, TR 25-50 ms, TE 20-30 ms); and (6) sub-millimetre 3D black-blood vessel-wall sequence (SPACE/VISTA/CUBE-VWI; TR 800–1000 ms, TE 20–30 ms) acquired on all 3 T systems.

Supplementary Method 2:Visual rating of cerebral small vascular disease burden and infarction determination

Visual rating of cerebral small vascular disease (CSVD)burden

1. White Matter Hyperintensities (WMH):

Fazekas Scale: We used the 3-point Fazekas scale to grade the severity of WMH based on axial T2-weighted fluid-attenuated inversion recovery sequences. This scale ranges from 0 (no WMH) to 3 (severe, confluent WMH extending into the deep white matter) [1] .

2. Lacunes:

3-Point Scale: Lacunes were assessed using a 3-point scale based on axial T2-weighted fluid-attenuated inversion recovery sequences, where Grade 0 indicates no lacunes, Grade 1 indicates 1 to 2 lacunes, and Grade 2 indicates 3 to 5 lacunes[1]. This grading system helps in quantifying the number and distribution of lacunes, which are small, round, or ovoid hypointensities typically less than 3 mm in diameter[2].

3. Microbleeds:

Presence or Absence: Microbleeds were identified using T2* gradient-recalled echo or susceptibility-weighted imaging sequences. We categorized the presence of microbleeds as either present or absent. Microbleeds appear as small, round, or quasi-round uniform low signals on SWI, with a diameter of 2–10 mm[2].

Imaging-Based Infarction Determination

MRI-based infarct identification was performed by trained raters using a standardized visual rating protocol based on predefined anatomical regions. The following infarct locations were systematically evaluated: left basal ganglia and surrounding white matter, angular gyrus, thalamus, basal forebrain, anterior cerebral artery (ACA) territory, posterior cerebral artery (PCA) territory, corpus callosum and infarction involving critical regions across the anterior cingulate and angular gyrus.

As most infarcts were chronic, primary identification was based on hyperintense lesions on fluid-attenuated inversion recovery (FLAIR) sequences, with supportive confirmation by

diffusion-weighted imaging (DWI) and apparent diffusion coefficient (ADC) maps when necessary. The minimum lesion size was 3 mm in diameter. Acute infarction was defined as a DWI-positive lesion acquired within 7 days of symptom onset.

For each patient, both the maximum infarct lesion size (in mm) and a qualitative classification (0 = 3–10 mm; 1 = >10 mm) were recorded. Lesion localization was guided by neuroanatomical landmarks on axial MRI slices and validated using multiplanar reconstructions. All imaging data were independently rated by two experienced reviewers blinded to clinical information, with discrepancies resolved by consensus.

Reference

1. Amin Al Olama A, Wason JMS, Tuladhar AM, et al. Simple MRI score aids prediction of dementia in cerebral small vessel disease. Neurology 2020;94(12):e1294-e1302. (In eng). DOI: 10.1212/wnl.0000000000009141.

2. Duering M, Biessels GJ, Brodtmann A, et al. Neuroimaging standards for research into small vessel disease-advances since 2013. Lancet Neurol 2023;22(7):602-618. (In eng). DOI: 10.1016/s1474-4422(23)00131-x.

Supplementary Table 1: Inter-rater agreement between two raters for ICAS and CSVD markers.

Marker	Scale	Metric	Estimate
ICA stenosis	Ordinal (no stenosis/ < 50% stenosis/ ≥ 50% stenosis)	Kappa	0.924
MCA stenosis	Ordinal (no stenosis/ < 50% stenosis/ ≥ 50% stenosis)	Kappa	0.882
ACA stenosis	Ordinal (no stenosis/ < 50% stenosis/ ≥ 50% stenosis)	Kappa	0.821
BA stenosis	Ordinal (no stenosis/ < 50% stenosis/ ≥ 50% stenosis)	Kappa	0.786
VA stenosis	Ordinal (no stenosis/ < 50% stenosis/ ≥ 50% stenosis)	Kappa	0.848
PCA stenosis	Ordinal (no stenosis/ < 50% stenosis/ ≥ 50% stenosis)	Kappa	0.999
Lacunar infarcts	Ordinal (grade 0/1/2/3)	Kappa	0.843
Fazekas score	Ordinal (grade 0/1/2/3)	Kappa	0.827
Cerebral microbleeds	Binary (present/ absent)	Kappa	0.800
CSVD burden	Continuous	ICC	0.814

Agreement for ordinal ratings was assessed using quadratically weighted Cohen's kappa. Agreement for the binary marker was assessed using unweighted Cohen's kappa. Because CSVD is a continuous measure, agreement was quantified using the two-way random-effects intraclass correlation coefficient for absolute agreement, single measurement.

Abbreviations: ICA, internal carotid artery; MCA, middle cerebral artery; ACA, anterior cerebral artery; BA, basilar artery; VA, vertebral artery; PCA, posterior cerebral artery; CSVD, cerebral small vessel disease; ICC, intraclass correlation coefficient.

Supplementary Table 2: Distribution of Enrolled Participants by Study Sites

	Group			
**center**	**Controls**	**aICAS <50%**	**aICAS ≥50%**	**sICAS ≥50%**
No.	98	84	154	165
Peking Union Medical College Hospital	77 (79%)	80 (95%)	135 (88%)	110 (67%)
The Third Affiliated Hospital of Guangzhou Medical University	0 (0%)	1 (1.2%)	1 (0.6%)	2 (1.2%)
West China Hospital	0 (0%)	0 (0%)	0 (0%)	4 (2.4%)
Liuzhou Traditional Chinese Medical Hospital	0 (0%)	0 (0%)	3 (1.9%)	18 (11%)
The General Hospital of the Chinese People's Liberation Army	2 (2.0%)	0 (0%)	2 (1.3%)	3 (1.8%)
Hebei General Hospital	12 (12%)	2 (2.4%)	7 (4.5%)	16 (9.7%)
Weihai Municipal Hospital	0 (0%)	0 (0%)	2 (1.3%)	7 (4.2%)
Shiyan Taihe Hospital	7 (7.1%)	1 (1.2%)	3 (1.9%)	4 (2.4%)
Dushu Lake Hospital Affiliated to Soochow University	0 (0%)	0 (0%)	1 (0.6%)	1 (0.6%)

Supplementary Table 3: Demographic factors and clinical data of participants （pairwise comparisons versus controls）

	Group							
	controls	aICAS <50%	aICAS ≥50%	sICAS ≥50%	P1	P2	P3	P4
No.	98	84	154	165				
Demographic factors								
Age, mean (SD), y	60.7(9.1)	57.7 (11.8)	58.8 (11.2)	61.3 (10.0)	0.079	0.122	0.180	0.567
Male, No.(%)	45 (46%)	42 (50%)	73 (47%)	109 (66%)	0.001	0.689	0.920	0.002
Education, mean (SD), y	12.61 (4.55)	13.10 (4.68)	12.00 (4.36)	10.62 (4.00)	<0.001	0.417	0.277	<0.001
Hypertension, No.(%)	32 (33%)	38 (45%)	85 (55%)	107 (65%)	<0.001	0.113	<0.001	<0.001
Diabetes mellitus, No.(%)	17 (17%)	21 (25%)	36 (23%)	65 (39%)	<0.001	0.279	0.324	<0.001
Hyperlipidemia, No.(%)	46 (47%)	49 (58%)	95 (62%)	103 (62%)	0.069	0.166	0.030	0.020
Atrial fibrillation, No.(%)	1 (1.0%)	1 (1.2%)	1 (0.6%)	1 (0.6%)	0.951	>0.999	>0.999	>0.999
Coronary disease, No.(%)	9 (9.2%)	8 (9.5%)	15 (9.7%)	22 (13%)	0.639	>0.999	>0.999	0.417
Smoking, No.(%)	24 (24%)	18 (21%)	37 (24%)	66 (40%)*	0.002	0.755	>0.999	0.015
Alcohol taken, No.(%)	50 (51%)	39 (46%)	69 (45%)	85 (52%)	0.607	0.639	0.404	>0.999
APOE ε4 carrier, No.(%)	18 (18%)	7 (8.3%)	28 (18%)	30 (18%)	0.175	0.081	>0.999	>0.999
ICAS characteristics								
Multiple vessels involved, No.(%)	0 (0%)	41 (49%)	103 (67%)	127 (77%)				
Posterior circulation involved, No.(%)	0 (0%)	32 (38%)	75 (49%)	103 (62%)				
Visible cerebral infarction lesions on MRI, No.(%)	7 (7.1%)	13 (15%)	29 (19%)	132 (80%)	<0.001	0.120	0.016	<0.001
CSVD burden, mean (SD)	0.96 (1.05)	1.35 (1.52)	1.41 (1.48)	2.63 (1.87)	<0.001	0.193	0.023	<0.001
Cognitive status								
MCI, No.(%)	22 (22%)	29 (35%)	59 (38%)	73 (44%)	<0.001	0.080	0.005	<0.001
Dementia, No.(%)	0 (0%)	2 (2.4%)	8 (5.2%)	32 (19%)	<0.001	0.340	0.026	<0.001
Any cognitive impairment, No.(%)	22 (22%)	31 (37%)	67 (43%)	105(63%)	<0.001	0.048	0.001	<0.001

P1: P-value for comparison of baseline characteristics across all 4 groups by Kruskal-Wallis test or χ^2 test. P2: P-value for comparison of baseline characteristics by Wilcoxon Rank Sum test or χ^2 test between aICAS <50% versus controls. P3: P-value for comparison of baseline characteristics by Wilcoxon Rank Sum test or χ^2 test between aICAS ≥50% versus controls. P4: P-value for comparison of baseline characteristics by Wilcoxon Rank Sum test or χ^2 test between sICAS ≥50% versus controls.

Abbreviation: ICAS, intracranial atherosclerosis; controls, ICAS-free controls; aICAS <50%, asymptomatic intracranial atherosclerosis with <50% stenosis; aICAS ≥50%, asymptomatic ICAS with ≥50% stenosis; sICAS ≥50%, symptomatic ICAS with ≥50% stenosis; CSVD, cerebral small vascular disease; SD, standard deviations; MCI, mild cognitive impairment.

Supplementary Table 4: Association of CSVD neuroimaging biomarkers with ICAS continuum

	Group						
	**Controls**	**aICAS <50%**	**aICAS <50%vs controls (β, 95% CI)**	**aICAS ≥50%**	**aICAS ≥50%vs controls (β, 95% CI)**	**sICAS ≥50%**	**sICAS ≥50%vs controls (β, 95% CI)**
CSVD score[a]	0.96 (1.05)	1.35 (1.52)	0.524 (0.098, 0.949)*	1.41 (1.48)	0.543 (0.174, 0.912)*	2.63 (1.87)	1.555 (1.188, 1.923)‡
lacunar infarcts ≥3, No.(%)[b]	2 (2.0%)	8 (9.5%)	5.627 (1.349, 38.246)*	13 (8.4%)	4.810 (1.281, 31.346)	53 (32%)	21.265 (6.327, 132.546)‡
Fazekas score ≥2, No.(%)[b]	11 (11%)	18 (21%)	2.590 (1.129, 6.182)*	29 (19%)	2.076 (0.987, 4.642)	60 (36%)	4.405 (2.206, 9.498)‡
Cerebral microbleeds (≥0)[b], No.(%)	8 (8.2%)	12 (14%)	2.048 (0.797, 5.527)	21 (14%)	1.892 (0.826, 4.746)	49 (30%)	4.481 (2.104, 10.727)‡

Abbreviation: ICAS, intracranial atherosclerosis; CSVD, cerebral small vascular disease; controls, ICAS-free controls; aICAS<50%, asymptomatic intracranial atherosclerosis with <50% stenosis; aICAS≥50%, asymptomatic intracranial atherosclerosis with ≥50% stenosis; sICAS≥50%, symptomatic intracranial atherosclerosis with ≥50% stenosis;

[a] Linear regression models assessed CSVD biomarkers (dependent variables) across ICAS continuum (aICAS<50%, aICAS ≥50%, sICAS ≥50%; as independent variables), adjusted for age, sex.

[b] Logistic regression models assessed lacunar infarcts, Fazekas score and Cerebral microbleeds （no/≥0) (dependent variables) across ICAS continuum (aICAS<50%, aICAS ≥50%, sICAS ≥50%; as independent variables), adjusted for age, sex.

* P< 0.05

† P< 0.001

‡ P< 0.001.

Supplementary Table 5: The neuropsychological test performance of participants (overall and prespecified pairwise comparisons)

	Group									
	Controls	aICAS <50%	aICAS ≥50%	sICAS ≥50%	P1	P2	P3	P4	P5	P6
No.	98	84	154	165						
MMSE, mean (SD)	27.35 (1.91)	26.71 (3.42)	26.34 (3.06)	25.12 (4.29)	<0.001	0.145	0.080	<0.001	>0.999	0.396
MOCA, mean (SD),	24.92 (3.10)	24.23 (4.16)	23.44 (4.38)	21.38 (5.41)	<0.001	0.098	0.012	<0.001	>0.999	0.062
Auditory Verbal Learning Test, total, mean (SD)	29.77 (8.93)	28.44 (11.00)	26.76 (11.49)	21.77 (11.11)	<0.001	0.172	0.031	<0.001	>0.999	0.193
Paired Association Learning Test, mean (SD)	12.19 (5.00)	11.40 (5.74)	10.10 (5.34)	9.05 (5.21)	0.002	0.338	0.003	0.003	>0.999	>0.999
Rey Complex Figure Recall, mean (SD)	20.48 (7.73)	19.09 (9.41	17.89 (8.55)	14.68 (9.07)	<0.001	0.272	0.054	<0.001	>0.999	0.260
Digit Span-forward, mean (SD)	7.78 (1.54)	8.12 (1.23)	7.87 (1.51)	7.12 (1.53)	0.002	>0.999	>0.999	0.137	>0.999	0.005
Digit Span-backward, mean (SD)	4.82 (1.55)	5.05 (1.71)	4.93 (1.60)	4.16 (1.58)	0.022	>0.999	>0.999	0.222	>0.999	0.020
Trail Making Test-A, mean (SD), seconds	64.98 (49.31)	59.72 (29.04)	59.66 (30.97)	76.78 (50.81)	0.053	>0.999	>0.999	0.990	>0.999	0.034
Trail Making Test-B, mean (SD), seconds	146.10 (58.32)	159.11 (71.33)	165.34 (84.75)	183.08 (92.62)	0.014	0.090	0.077	0.013	>0.999	>0.999
Clock Drawing Test, mean (SD)	2.68 (0.57)	2.61 (0.64)	2.58 (0.68)	2.24 (0.88)	<0.001	>0.999	>0.999	<0.001	>0.999	0.005
Symbol Digit Modalities Test, mean (SD)	42.06 (14.66)	42.22 (16.84)	41.27 (16.26)	31.11 (16.84)	<0.001	0.594	>0.999	<0.001	>0.999	<0.001
Rey Complex Figure Copy, mean (SD)	34.22 (4.00)	33.94 (4.86)	33.21 (6.02)	30.97 (8.31)	0.017	>0.999	>0.999	0.015	>0.999	0.203
Boston Naming Test, mean (SD)	26.09 (3.04)	25.46 (4.08)	24.68 (3.93)	23.98 (4.47)	0.009	0.335	0.028	0.007	>0.999	>0.999
Verbal Fluency, naming animal, mean (SD)	19.83 (5.07)	20.11 (7.83)	18.84 (6.70)	15.30 (6.40)	<0.001	>0.999	>0.999	<0.001	>0.999	0.003
Calculation, mean (SD)	11.82 (3.73)	11.17 (3.73)	10.47 (3.72)	9.55 (3.83)	<0.001	0.089	0.007	<0.001	>0.999	>0.999

P1: P-value for comparison of baseline characteristics across all 4 groups by ANOVA. P2: P-value for comparison of baseline characteristics by ANOVA between aICAS <50% versus controls. P3: P-value for comparison of baseline characteristics by ANOVA between aICAS ≥50% versus controls. P4: P-value for comparison of baseline characteristics by ANOVA between sICAS ≥50% versus controls. P5: P-value for comparison of baseline characteristics by ANOVA between aICAS ≥50% versus aICAS <50%. P6: P-value for comparison of baseline characteristics by ANOVA between sICAS ≥50% versus aICAS ≥50%. All comparison were adjusted with age, sex and education. Abbreviation: ICAS, intracranial atherosclerosis; controls, ICAS-free controls; aICAS <50%, asymptomatic intracranial atherosclerosis with <50% stenosis; aICAS ≥50%, asymptomatic ICAS with ≥50% stenosis; sICAS ≥50%, symptomatic ICAS with ≥50% stenosis; SD, standard deviations.

Supplementary Table 6: Multivariable logistic regression analysis of ICAS severity and plasma biomarkers in relation to cognitive impairment .

	No.	OR (95% CI)	Wald z	P value
Model A[a]				
controls	98	1 [reference]		
aICAS < 50%	84	2.54 (1.24, 5.29)	6.38	0.012
aICAS ≥ 50%	154	2.77 (1.50, 5.26)	10.18	0.001
sICAS ≥ 50%	165	4.88 (2.62, 9.35)	24.06	<0.001
Model B[b]				
Aβ42/ Aβ40, ln	501	0.64 (0.22, 1.87)	0.65	0.419
pTau217	501	2.27 (0.48, 11.30)	1.05	0.306
GFAP	501	1.00 (1.00, 1.01)	0.08	0.780
NfL	501	1.01 (1.00, 1.03)	2.07	0.150
Model C[c]				
controls	98	1 [reference]		
aICAS < 50%	84	2.48 (1.20, 5.22)	5.91	0.015
aICAS ≥ 50%	154	2.65 (1.41, 5.08)	8.93	0.003
sICAS ≥ 50%	165	4.14 (2.10, 8.37)	16.28	<0.001
Aβ42/ Aβ40, ln	501	0.71 (0.23, 2.14)	0.37	0.545
pTau217	501	1.67 (0.29, 9.96)	0.33	0.566
GFAP	501	1.00 (0.99, 1.01)	0.14	0.710
NfL	501	1.01 (0.99, 1.03)	1.40	0.236
CSVD	501	1.04(0.90, 1.19)	0.23	0.633

Multivariable logistic regression models showing adjusted odds ratios (ORs) and 95% confidence intervals (CIs) for the association between ICAS, plasma biomarkers, and cognitive impairment (including mild cognitive impairment or dementia). ICAS was associated with significantly higher odds of cognitive impairment in a graded manner across all models. In contrast, none of the plasma biomarkers showed a significant association with cognitive impairment.

Model A included ICAS severity and adjusted for age, sex, education level, study center, hypertension, diabetes mellitus, smoking status, and APOE ε4 carrier status.

Model B additionally included each plasma biomarker separately (natural log–transformed Aβ42/40 ratio, pTau217, GFAP, or NfL) with the same adjustments as Model A.

Model C simultaneously included ICAS severity, all four plasma biomarkers (ln-transformed Aβ42/40 ratio, pTau217, GFAP, and NfL), and CSVD burden, with adjustments identical to Model A.

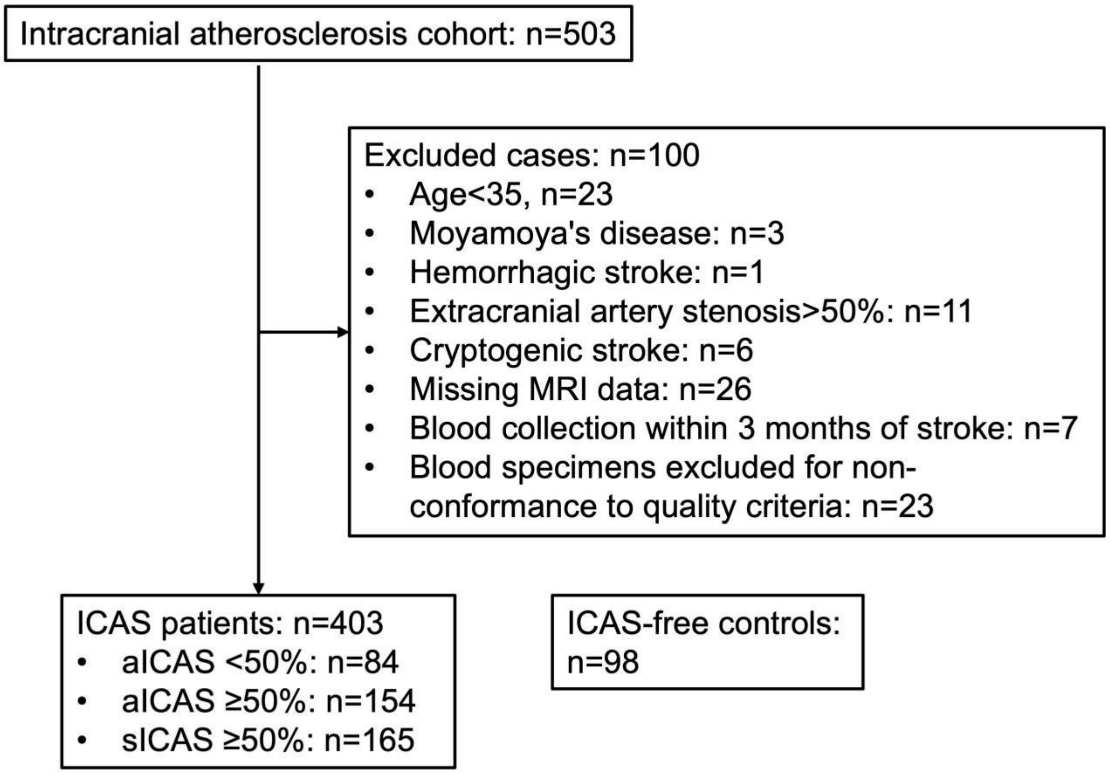

Supplementary Figure 1: Flow diagram of the recruitment of study participants

	No.	OR (95% CI)	*P* value
**Age < 60y**			
controls	41	1 [reference]	
aICAS < 50%	43	4.01 (1.10, 15.9)	0.040
aICAS ≥ 50%	79	3.99 (1.29, 13.7)	0.021
sICAS ≥ 50%	66	4.16 (1.19, 15.8)	0.030
Aβ42/ Aβ40, ln	229	0.24 (0.03, 1.54)	0.131
pTau217	229	1.91 (0.02, 138.68)	0.769
GFAP	229	1.01 (1.00, 1.03)	0.068
NfL	229	1.00 (0.97, 1.02)	0.887
**Age ≥ 60y**			
controls	57	1 [reference]	
aICAS < 50%	41	2.34 (0.94, 5.97)	0.070
aICAS ≥ 50%	76	2.55 (1.15, 5.87)	0.024
sICAS ≥ 50%	100	6.15 (2.60, 15.3)	<0.001
Aβ42/ Aβ40, ln	272	1.48 (0.35, 6.57)	0.596
pTau217	272	4.66 (0.57, 41.80)	0.156
GFAP	272	0.99 (0.98, 1.00)	0.101
NfL	272	1.02 (1.00, 1.06)	0.092
**Male**			
controls	45	1 [reference]	
aICAS < 50%	42	2.24 (0.78, 6.70)	0.140
aICAS ≥ 50%	73	2.70 (1.07, 7.19)	0.040
sICAS ≥ 50%	109	4.82 (1.89, 13.1)	0.001
Aβ42/ Aβ40, ln	269	0.81 (0.18, 3.53)	0.778
pTau217	269	0.87 (0.05, 14.10)	0.921
GFAP	269	1.00 (0.99, 1.01)	0.951
NfL	269	1.02 (1.00, 1.04)	0.140
**Female**			
controls	53	1 [reference]	
aICAS < 50%	42	3.07 (1.10, 8.90)	0.034
aICAS ≥ 50%	81	2.53 (1.04, 6.45)	0.045
sICAS ≥ 50%	56	3.68 (1.28, 10.90)	0.017
Aβ42/ Aβ40, ln	232	0.61 (0.10, 3.56)	0.587
pTau217	232	2.94 (0.25, 36.90)	0.392
GFAP	232	1.00 (0.99, 1.01)	0.752
NfL	232	1.00 (0.97, 1.03)	0.931

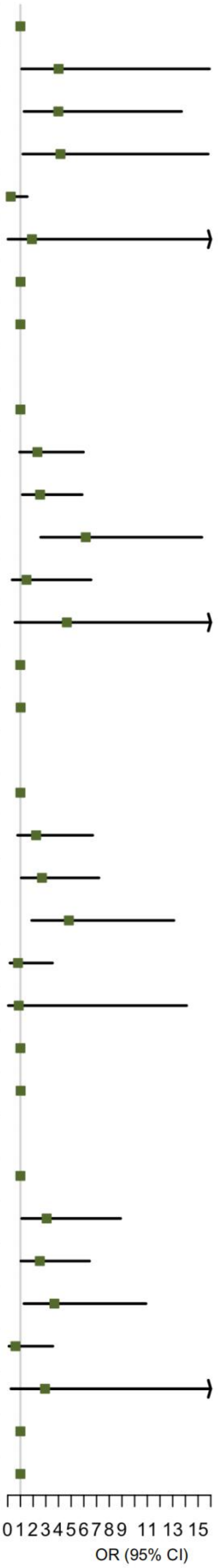

Supplementary Figure 2: Stratified associations of ICAS severity with cognitive impairment.

Forest plots show adjusted odds ratios (ORs) with 95% confidence intervals (CIs) and two-sided p values for the presence of any cognitive impairment (mild cognitive impairment or dementia), stratified by age (<60 years; ≥60 years) (A) and sex (male; female) (B). Controls (ICAS-free) served as the reference category for ICAS severity. No. indicates the number of participants included in each category within the corresponding stratum.

Statistical analysis. Within each stratum, associations were assessed using multivariable binary logistic regression including ICAS severity, all four plasma biomarkers (ln-transformed Aβ42/40 ratio, pTau217, GFAP, and NfL), and CSVD burden as independent variables. Models were adjusted for years of education, study centre, hypertension, diabetes mellitus, smoking status, and APOE ε4 carrier status, and additionally for sex (in age-stratified models) or age (in sex-stratified models). p values were derived from Wald tests for regression coefficients; corresponding Wald z statistics for the ICAS contrasts (vs controls) are as follows:

Age <60 years: aICAS<50%, z=4.20, P=0.040; aICAS≥50%, z=5.36, P=0.021; sICAS≥50%, z=4.73, P=0.030;

Age ≥60 years: aICAS<50%, z=3.29, P=0.070; aICAS≥50%, z=5.09, P=0.024; sICAS≥50%, z=16.25, P<0.001;

Male: aICAS<50%, z=2.17, P=0.140; aICAS≥50%, z=4.23, P=0.040; sICAS≥50%, z=10.30, P=0.001;

Female: aICAS<50%, z=4.50, P=0.034; aICAS≥50%, z=4.04, P=0.045; sICAS≥50%, z=5.72, P=0.017.

Statistical significance was defined as two-sided P<0.05; no adjustment for multiple comparisons was applied. Biomarker coefficients are shown for completeness; none were significantly associated with cognitive impairment in these stratified models

Experimental unit. The experimental unit was the individual participant (one cognitive outcome classification per participant; biomarker values measured per participant).

Abbreviations: ICAS, intracranial atherosclerosis; aICAS, asymptomatic ICAS; sICAS, symptomatic ICAS; Aβ, amyloid-β; pTau217, phosphorylated tau (threonine 217); GFAP, glial fibrillary acidic protein; NfL, neurofilament light chain; CSVD, cerebral small vessel disease.

Total	No.	OR (95% CI)	*P* value
**Model A**[a]			
controls	91	1 [reference]	
aICAS < 50%	71	2.85 (1.25, 6.67)	0.014
aICAS ≥ 50%	125	3.50 (1.74, 7.37)	<0.001
**Model B**[b]			
Aβ42/ Aβ40, ln	287	0.41 (0.09, 1.99)	0.267
pTau217	287	2.85 (0.33, 25.70)	0.341
GFAP	287	1.00 (0.99, 1.01)	0.822
NfL	287	1.03 (0.98, 1.09)	0.310
**Model C**[c]			
controls	91	1 [reference]	
aICAS < 50%	71	2.84 (1.23, 6.76)	0.016
aICAS ≥ 50%	125	3.38 (1.64, 7.29)	0.001
Aβ42/ Aβ40, ln	287	0.47 (0.09, 2.53)	0.374
pTau217	287	2.79 (0.25, 31.80)	0.402
GFAP	287	0.99 (0.98, 1.00)	0.263
NfL	287	1.03 (0.98, 1.10)	0.264

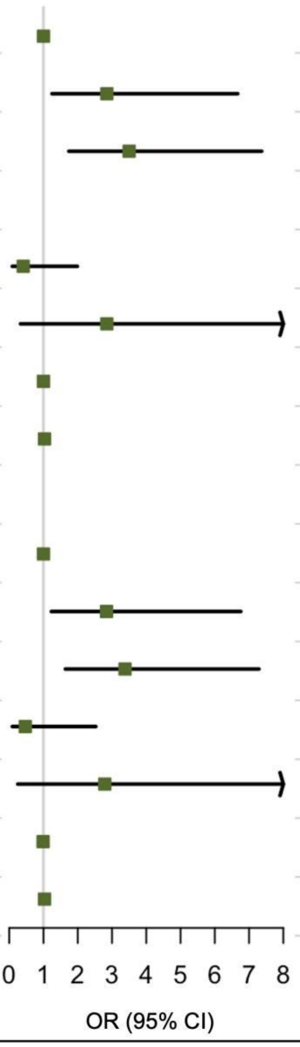

Supplementary Figure 3: Association between asymptomatic intracranial atherosclerosis (aICAS) and cognitive impairment after excluding participants with silent infarcts.

Forest plot shows adjusted odds ratios (ORs) with 95% confidence intervals (CIs) and two-sided p values from multivariable binary logistic regression models for any cognitive impairment (mild cognitive impairment or dementia) after excluding participants with silent infarcts. Controls (ICAS-free) served as the reference category for aICAS severity. No. indicates the number of participants included in each category (and the number with available biomarker data for continuous terms).

Statistical analysis. p values were obtained from Wald tests of logistic-regression coefficients (Wald z/χ^2 statistics). Model A included aICAS severity and covariates. Model B additionally included each plasma biomarker separately [ln(Aβ42/40), pTau217, GFAP, or NfL] with the same covariate adjustment. Model C simultaneously included aICAS severity, all four biomarkers, and CSVD burden, with covariate adjustment identical to Model A. Covariates were age, sex, years of education, study centre, hypertension, diabetes mellitus, smoking status, and APOE ε4 carrier status. Statistical significance was defined as two-sided $p<0.05$; no adjustment for multiple comparisons was applied.

Experimental unit. The experimental unit was the individual participant.

Abbreviations: aICAS, asymptomatic intracranial atherosclerosis; ICAS, intracranial atherosclerosis; Aβ, amyloid-β; pTau217, phosphorylated tau (threonine 217); GFAP, glial fibrillary acidic protein; NfL, neurofilament light chain; CSVD, cerebral small vessel disease.

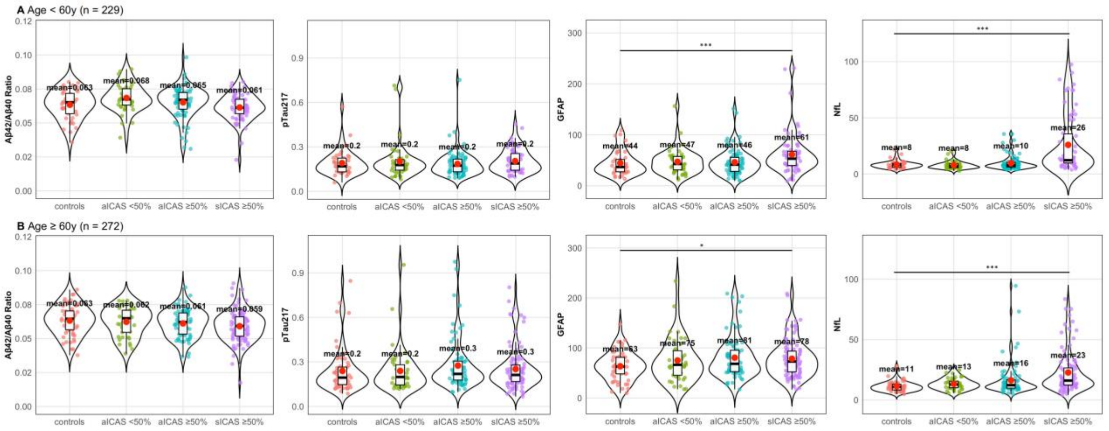

Supplementary Figure 4: Plasma biomarkers by intracranial atherosclerosis (ICAS) severity (age-stratified analyses).

(A) Among participants aged ≥60 years, the Aβ42/40 ratio showed a trend toward reduction in the sICAS≥50% group (β= −0.004, 95% CI −0.007 to 0.000, t= -1.95, P= 0.052), whereas pTau217 remained unchanged (β= 0.01, 95% CI −0.04 to 0.06, t= 0.24, P= 0.884). GFAP was borderline elevated in the aICAS≥50% group (β= 12.15, 95% CI −0.01 to 24.31, t= 1.97, P= 0.050) and was significantly elevated in the sICAS≥50% group (β= 14.39, 95% CI 2.88 to 25.89, t= 2.46, P= 0.014). NfL was significantly elevated in the sICAS≥50% group (β= 10.80, 95% CI 6.61 to 15.00, t= 5.07, P< 0.001).

(B) Among participants aged <60 years, results were generally consistent with those observed in the ≥60-year subgroup. GFAP was significantly elevated in the sICAS≥50% group (β=19.58, 95% CI 7.51 to 31.65, t= 3.20, P= 0.002).NfL was significantly elevated in the sICAS≥50% group (β=16.70, 95% CI 10.94 to 22.46, t= 5.71, P< 0.001).

Statistical analysis. Within each age stratum, associations between ICAS severity (categorical; reference group: controls) and plasma biomarker levels were assessed using multivariable linear regression for each biomarker (dependent variables: natural log–transformed Aβ42/40 ratio, pTau217, GFAP, and NfL), adjusting for age (as applicable within stratum), sex, and APOE ε4 carrier status. For each group contrast versus controls, results are reported as adjusted regression coefficients (β) with

95% confidence intervals, together with a uniform nominal significance threshold of $P < 0.05$ across all analyses.

Experimental unit. The experimental unit was the individual participant (one cognitive outcome classification per participant; biomarker levels measured per participant).

Abbreviations: ICAS, intracranial atherosclerosis; aICAS, asymptomatic ICAS; sICAS, symptomatic ICAS; Aβ, amyloid-β; pTau217, phosphorylated tau (threonine 217); GFAP, glial fibrillary acidic protein; NfL, neurofilament light chain.
